# Aphids (Hemiptera, Aphididae) on ornamental plants in greenhouses in Bulgaria

**DOI:** 10.3897/zookeys.319.4318

**Published:** 2013-07-30

**Authors:** Mariya Yovkova, Olivera Petrović-Obradović, Elena Tasheva-Terzieva, Aneliya Pencheva

**Affiliations:** 1 Faculty of Ecology and Landscape Architecture, University of Forestry, 10 Kliment Ohridski Blvd, 1756 Sofia, Bulgaria; 2 Faculty of Agriculture, University of Belgrade, 6 Nemanjina str., 11080 Beograd–Zemun, Serbia; 3 Faculty of Biology, Sofia University “St. Kliment Ohridski”, 8 Dragan Tsankov Blvd, 1164 Sofia, Bulgaria

**Keywords:** Aphididae, aphids, ornamental plants, greenhouses, Bulgaria

## Abstract

Investigations on the species composition and host range of aphids on ornamental greenhouse plants in Bulgaria was conducted over a period of five years, from 2008 to 2012. Twenty greenhouses, growing ornamentals for landscaping, plant collections and other purposes were observed. They were located in the regions of Sofia, Plovdiv, Smolyan, Pavlikeni, Varna and Burgas. The total number of collected aphid samples was 279. Their composition included 33 aphid species and one subspecies from 13 genera and 5 subfamilies. Twenty-eight species were found to belong to subfamily Aphidinae. Almost 70 % of all recorded species were polyphagous. The most widespread aphid species was *Myzus persicae*, detected in 13 greenhouses all year round, followed by *Aulacorthum solani* (10 greenhouses) and *Aphis gossypii* (9 greenhouses). The widest host range was shown by *Myzus persicae* (43 hosts), *Aulacorthum solani* (32 hosts) and *Aulacorthum circumflexum* (23 hosts).

The list of host plants includes 114 species from 95 genera and 58 families. The greatest variety of aphid species was detected on *Hibiscus* (9 species). Out of all aphid samples 12.9 % were collected on *Hibiscus* and 6.8 %, on *Dendranthema*. The greatest variety of aphid species was detected on *Hibiscus* (9 species).

*Periphyllus californiensis* and *Aphis (Aphis) fabae mordvilkoi* are reported for the first time for Bulgaria. Furthermore, *Aphis spiraecola* has been found in new localities and has widened its host range in this country.

## Introduction

Aphids cause serious damage in greenhouses, where conditions are favorable for their development throughout the year and where they can reach high density over a short period of time. The damage caused by aphids may lead to deterioration of the ornamental qualities of infested plants and sometimes even death. One of the most important and serious consequences is virus transmission.

Currently, there is no extensive research on ornamental plants in greenhouses in Bulgaria, which prompted the current study. The only survey on this topic was conducted by Tashev (1962). He reported 6 aphid species: *Aulacorthum (Neomyzus) circumflexum* (Buckton, 1876); *Macrosiphoniella sanborni* (Gillette, 1908); *Macrosiphum euphorbiae* (Thomas, 1878); *Myzus (Myzus) ornatus* Laing, 1932; *Myzus (Nectarosiphon) ascalonicus* Doncaster, 1946; *Myzus (Nectarosiphon) persicae* (Sulzer, 1776).

The results of our survey contribute to the scientific knowledge in the investigated field, but also have a practical application, benefitting producers of ornamental crops. The purpose of this study was to identify the species composition of aphids on greenhouse ornamentals, their host range and the most frequently infested ornamental species in Bulgaria.

## Material and methods

The investigation was conducted over a period of five years, from 2008 to 2012. Twenty greenhouses, located in the regions of Sofia, Plovdiv, Smolyan, Pavlikeni, Varna and Burgas, were observed. Several types of greenhouses were included: for growing and propagation of ornamentals, for landscaping (annuals, perennials, rooting cuttings), for acclimatization of imported plants, for winter preservation of cold-tender species and for plant collections.

The observed greenhouses are designated as follows:

Greenhouses with a permanent plant composition: Greenhouses of University of ForestryGL; Greenhouses of Bulgarian Academy of ScienceGB; Greenhouses of the University Botanic GardensGS; Greenhouses of Euxinograd parkGE; Greenhouses of Vrana parkGV; Greenhouses of Krichim parkGK; RavdaR1;

Greenhouses with a constant circulation of plant species: private greenhouses in Sofia (S1, S2, S3, S4, S5, S6); Varna (V1, V2); Burgas (B1); Ravda (R2); Pavlikeni (PV); Plovdiv (PL); Smolyan (SM).

Aphids were collected in plastic bags together with the infested plant parts. Larvae were reared in laboratory conditions to the stage of adults.

The species identification was carried out using permanent microscope slides, after the traditional method of Hille Ris Lambers (1950). Identification keys included Blackman and Eastop (1994, 2000, 2006), Shaposhnikov (1964) and Stroyan (1984).

## Results

The total number of collected samples of aphids on ornamental plants during the observed five-year period was 279.

In total, 33 aphid species and one subspecies from 13 genera and 5 subfamilies (Lachninae, Chaitophorinae, Calaphidinae, Aphidinae and Eriosomatinae) were identified. Four species were identified only to the generic level but they are included in a total number of species because of the presence of clear distinguishing characters proving that they are separate species. Fourteen species belong to genus *Aphis* (42 %) and three species belong to genus *Myzus* (9 %). Genera *Aulacorthum*, *Brachycaudus*, *Macrosiphum* and *Rhopalosiphum* are represented by 2 species. Genera *Cinara*, *Idiopterus*, *Macrosiphoniella*, *Ovatus*, *Periphyllus*, *Prociphilus* and *Tinocallis* are represented by 1 species.

All aphid species and their host plants, including the data of Tashev (1962), are presented in alphabetical order in Table 1.

**Table 1.** Aphid species on greenhouse ornamental host plants recorded in Bulgaria.

**Aphid species**	**Host plant family**	**Host plant species**	**Greenhouse**	**Date**
*Aphis (Aphis) craccivora* Koch, 1854	Fabaceae	*Robinia pseudoacacia* L.	GL	27.07.2010
		*Wisteria chinensis* Siebold	R1	17.07.2009
	Malvaceae	*Hibiscus rosa-sinensis* L.	GL	11.07.2011
	Nyctaginaceae	*Bougainvillea glabra* Choisy	GE	27.05.2009
	Portulacaceae	*Portulaca umbraticola* Kunth	B1	17.08.2010
*Aphis (Aphis) fabae* Scopoli, 1763*	Agavaceae	*Yucca elephantipes* Hort. ex Regel	S1	05.07.2011
	Aizoaceae	*Aptenia cordifolia* (L.f.) Schwantes	GL	18.09.2009
				30.06.2009
	Araceae	*Anthurium andraeanum* Linden	GE	01.06.2010
	Asteraceae	*Cosmos bipinnatus* Cav.	GL	11.07.2011
	Nyctaginaceae	*Bougainvillea glabra* Choisy	GE	13.07.2009
	Solanaceae	*Datura hybrida* Ten.	GE	27.05.2009
*Aphis (Aphis)fabae mordvilkoi* Börner & Janich, 1922	Tropaeolaceae	*Tropaeolum majus* L.	GE	01.06.2010
*Aphis (Aphis)* ex gr. *fabae*	Araceae	*Anthurium andraeanum* Linden	GE	01.06.2010
*Aphis (Aphis) farinosa* Gmelin, 1790	Salicaceae	*Salix matsudana* Koidz.	GB	26.05.2010
*Aphis (Aphis)* ex gr. *frangulae*	Asteraceae	*Gazania heterophylla* Willd. ex Steud.	S2	06.08.2010
*Aphis (Aphis) gossypii* Glover, 1877	Acanthaceae	*Aphelandra squarrosa* Nees	S1	13.03.2009
				18.03.2009
				26.05.2010
			S3	28.04.2010
	Araliaceae	*Schefflera arboricola* (Hayata) Merr.	GS	05.03.2010
	Asteraceae	*Chrysanthemum hybridum* Guss.	S1	17.02.2009
				25.11.2008
		*Dendranthema* sp.	S1	13.02.2009
				13.03.2009
				25.11.2008
			S6	26.11.2009
	Malvaceae	*Hibiscus rosa-sinensis* L.	GL	05.11.2010
				08.10.2010
				11.07.2011
				18.09.2009
				20.07.2011
				31.08.2010
			GV	28.10.2008
			R1	01.06.2010
				19.07.2010
		*Hibiscus syriacus* L.	GB	04.08.2010
			GL	20.08.2010
				30.07.2010
	Primulaceae	*Cyclamen persicum* Mill.	S4	03.08.2010
	Rosaceae	*Eriobotrya japonica* (Thunb.) Lindl.	R1	17.07.2009
	Scrophulariaceae	*Hebe* sp.	S1	01.07.2010
*Aphis (Aphis) hederae* Kaltenbach, 1843	Araliaceae	*Hedera helix* L.	GE	23.07.2010
			S2	06.08.2010
				12.07.2011
				27.05.2010
*Aphis (Aphis) nasturtii* Kaltenbach, 1843	Malvaceae	*Hibiscus rosa-sinensis* L.	GL	05.11.2010
				31.08.2010
		*Hibiscus syriacus* L.	GL	31.08.2010
			R1	20.08.2010
	Primulaceae	*Cyclamen persicum* Mill.	S4	03.08.2010
*Aphis (Aphis) nerii* Boyer de Fonscolombe, 1841	Apocynaceae	*Nerium oleander* L.	R1	17.07.2009
				19.07.2010
	Asclepiadaceae	*Asclepias curassavica* L.	R1	17.07.2009
*Aphis (Aphis) sedi* Kaltenbach, 1843	Crassulaceae	*Sedum adolphi* Raym.-Hamet	GE	27.05.2009
		*Sedum glaucophyllum* R. T. Clausen	GE	27.05.2009
*Aphis (Aphis) spiraecola* Patch, 1914	Aizoaceae	*Aptenia cordifolia* (L.f.) Schwantes	GL	14.07.2010
				31.08.2010
	Caprifoliaceae	*Viburnum tinus* L.	R1	01.06.2010
	Malvaceae	*Hibiscus rosa-sinensis* L.	GL	05.11.2010
				08.10.2010
	Rosaceae	*Aronia melanocarpa* Nutt. ex Elliott	R1	17.07.2009
				29.05.2009
		*Spiraea douglasii* Hook.	PV	14.07.2010
		*Spiraea japonica* L. f.	PV	14.07.2010
		*Spiraea vanhouttei* (Briot) Carrière	PV	14.07.2010
	Scrophulariaceae	*Hebe* sp.	S1	01.07.2010
*Aphis (Aphis) spiraephaga* F. P. Müller, 1961	Rosaceae	*Spiraea douglasii* Hook.	S2	06.08.2010
*Aphis (Aphis) verbasci* Schrank, 1801	Buddlejaceae	*Buddleja davidii* Franch.	GB	04.08.2010
*Aphis (Aphis)* sp.	Begoniaceae	*Begonia semperflorens* Hook.	PL	19.08.2010
*Aulacorthum (Aulacorthum) solani* (Kaltenbach, 1843)	Acanthaceae	*Aphelandra squarrosa* Nees	R2	01.06.2010
			S1	28.04.2010
	Aceraceae	*Acer palmatum* Thunb.	GB	05.11.2010
	Apocynaceae	*Mandevilla sanderii* (Hemsl.) Woodson	S5	12.07.2011
		*Nerium oleander* L.	GL	08.06.2011
		*Vinca major* L.	GL	08.06.2011
				28.03.2011
	Araceae	*Anthurium andraeanum* Linden	GE	23.07.2010
			S1	28.04.2010
		*Syngonium podophyllum* Schott	GL	08.06.2011
		*Syngonium* sp.	R2	01.06.2010
	Araliaceae	*Aralia japonica* Thunb.	GL	14.03.2012
		*Hedera helix* L.	GL	28.03.2011
		*Schefflera arboricola* (Hayata) Merr.	GL	08.06.2011
			R1	01.06.2010
*Aulacorthum (Aulacorthum) solani* (Kaltenbach, 1843)	Asteraceae	*Chrysanthemum hybridum* Guss.	S4	12.07.2011
		*Dahlia x cultorum* Thorsrud & Reisaeter	S5	26.05.2010
		*Dendranthema* sp.	S4	12.07.2011
		*Gerbera jamesonii* Adlam	GL	08.06.2011
		*Senecio macroglossus* DC.	GV	28.10.2008
	Begoniaceae	*Begonia elatior* Hort. ex Steud.	S5	01.07.2010
	Caesalpiniaceae	*Gleditsia triacanthos* L.	GL	08.06.2011
	Caprifoliaceae	*Weigela floribunda* C. A. Mey	R1	01.06.2010
		*Weigela florida* A. DC.	GL	08.06.2011
	Geraniaceae	*Pelargonium peltatum* (L.) L’Hèr.	GL	05.11.2010
				14.03.2012
				28.03.2011
			S2	19.05.2011
		*Pelargonium roseum* Ehrh.	GL	08.06.2011
		*Pelargonium zonale* (L.) L’Hèr.	GL	01.04.2010
				05.11.2010
				14.07.2010
				18.09.2009
				21.03.2011
				27.04.2010
			S2	19.05.2011
				24.01.2011
	Lamiaceae	*Coleus x hybridus* Hort.	GL	08.06.2011
		*Mentha* sp.	GB	05.11.2010
		*Thymus* sp.	GB	05.11.2010
	Lauraceae	*Persea americana* Mill.	GL	08.06.2011
	Liliaceae	*Tulipa* sp.	S5	26.05.2010
	Malvaceae	*Hibiscus rosa-sinensis* L.	GB	07.10.2010
			GL	08.06.2011
				11.07.2011
				28.03.2011
				28.05.2011
	Onagraceae	*Fuchsia hybrida* Hort.	GL	08.06.2011
	Solanaceae	*Calibrachoa* sp.	S5	26.05.2010
				28.04.2010
	Verbenaceae	*Verbena x hybrida* Hort. ex Vilm.	S5	26.05.2010
*Aulacorthum (Neomyzus) circumflexum* (Buckton, 1876)	Acanthaceae	*Acanthus* sp.	GB	Tashev (1962)
		*Ruellia speciosa* Lindau	GB	Tashev (1962)
	Adiantaceae	*Adianthum capillus-veneris* L.	GL	21.03.2011
	Amaryllidaceae	*Nerine* sp.	GB	Tashev (1962)
	Anthericaceae	*Chlorophytum comosum* (Thunb.) Jacques	GL	17.02.2009
				26.05.2010
*Aulacorthum (Neomyzus) circumflexum* (Buckton, 1876)	Apocynaceae	*Catharanthus* sp.	GL	28.03.2011
		*Vinca major* L.	GL	01.04.2010
				08.06.2011
				11.07.2011
	Araceae	*Alocasia macrorrhizos* (L.) G. Don	GB	Tashev (1962)
			GL	28.03.2011
		*Anthurium andraeanum* Linden	GE	30.06.2009
		*Calla* sp.	GB	Tashev (1962)
		*Colocasia antiquorum* Schott	GB	Tashev (1962)
		*Syngonium podophyllum* Schott	GL	01.04.2010
				03.12.2008
				17.02.2009
				28.03.2011
				30.06.2009
		*Zantedeschia aethiopica* (L.) Spreng.	GL	08.06.2011
	Asteraceae	*Chrysanthemum hybridum* Guss.	S5	28.04.2010
		*Chrysanthemum indicum* L.	GB	Tashev (1962)
		*Cineraria* sp.	GB	Tashev (1962)
		*Dendranthema* sp.	S5	28.04.2010
		*Gerbera jamesonii* Adlam	GL	28.03.2011
		*Tagetes patula* L.	GL	21.03.2011
	Begoniaceae	*Begonia semperflorens* Hook.	GL	28.03.2011
	Bombacaceae	*Ceiba pentandra* Gaertn.	GL	21.03.2011
				28.03.2011
	Commelinaceae	*Tradescantia* sp.	GB	Tashev (1962)
	Corylaceae	*Carpinus betulus* L.	GL	08.06.2011
	Ericaceae	*Erica arborea* L.	GB	Tashev (1962)
	Hyacinthaceae	*Scilla maritima* L.	GB	Tashev (1962)
		*Scilla peruviana* L.	GB	Tashev (1962)
	Hydrocharitaceae	*Hydrilla verticillata* (L.f.) Royle	GB	Tashev (1962)
	Iridaceae	*Tritonia fenestrata* Ker Gawl.	GB	Tashev (1962)
	Lamiaceae	*Coleus x hybridus* Hort.	GB	Tashev (1962)
	Malvaceae	*Abutilon hybridum* Hort.	GL	08.06.2011
			GB	Tashev (1962)
		*Hibiscus rosa-sinensis* L.	GL	03.12.2008
				30.06.2009
*Aulacorthum (Neomyzus) circumflexum* (Buckton, 1876)	Nymphaeaceae	*Nymphaea coerulea* Lam.	GB	Tashev (1962)
		*Nymphaea* sp.	GB	Tashev (1962)
	Oxalidaceae	*Oxalis floribunda* Lehm.	GB	Tashev (1962)
		*Oxalis* sp.	GL	28.03.2011
			GB	Tashev (1962)
		*Oxalis violacea* L.	GB	Tashev (1962)
	Polypodiaceae	*Polypodium vulgare* L.	GB	Tashev (1962)
	Primulaceae	*Cyclamen persicum* Mill.	GL	28.03.2011
		*Cyclamen* sp.	GB	Tashev (1962)
		*Primula obconica* Hance	GL	28.03.2011
	Ranunculaceae	*Aquilegia vulgaris* L.	GL	08.06.2011
	Rosaceae	*Aronia melanocarpa* Nutt. ex Elliott	GL	08.06.2011
	Rubiaceae	*Hoffmannia refulgens* Hemsl.	GE	23.07.2010
	Salviniaceae	*Salvinia auriculata* Aubl.	GB	Tashev (1962)
	Saxifragaceae	*Saxifraga sarmentosa* L.f.	GB	Tashev (1962)
	Tiliaceae	*Sparmannia palmata* Hort. ex Lindl.	GB	Tashev (1962)
*Brachycaudus (Acaudus) cardui* (Linnaeus, 1758)	Asteraceae	*Chrysanthemum frutescens* L.	GB	05.11.2010
		*Dahlia x cultorum* Thorsrud & Reisaeter	SM	05.09.2011
		*Senecio cineraria* DC.	GL	08.06.2011
				11.07.2011
				28.03.2011
*Brachycaudus (Brachycaudus) helichrysi* (Kaltenbach, 1843)	Asteraceae	*Senecio mikanioides* Otto ex Walp.	GB	04.08.2010
*Cinara (Cinara) neubergi* (Arnhart, 1930)	Pinaceae	*Pinus pinaster* Aiton	R1	29.05.2009
*Idiopterus nephrelepidis* Davis, 1909	Adiantaceae	*Adianthum capillus-veneris* L.	S1	25.05.2011
	Araceae	*Syngonium podophyllum* Schott	GL	23.03.2011
	Aspleniaceae	*Asplenium nidus* L.	V1	13.07.2009
	Blechnaceae	*Blechnum* sp.	S1	25.05.2011
	Davalliaceae	*Nephrolepis exaltata* (L.) Schott	GL	25.04.2012
				30.03.2012
			S1	13.03.2009
				25.05.2011
			S3	06.08.2010
				19.05.2011
	Piperaceae	*Peperomia clusiifolia* Hook.	GL	28.03.2011
	Polypodiaceae	*Platycerium bifurcatum* (Cav.) C. Chr.	V1	13.07.2009
*Idiopterus nephrelepidis* Davis, 1909	Pteridaceae	*Pteris cretica* L.	GL	28.03.2011
		*Pteris* sp.	S1	25.05.2011
*Macrosiphoniella (Macrosiphoniella) sanborni* (Gillette, 1908)	Asteraceae	*Chrysanthemum hybridum* Guss.	S1	20.10.2010
			S4	12.07.2011
			S5	07.10.2010
				18.12.2008
				26.05.2010
				28.04.2010
			S6	01.11.2011
				26.11.2009
		*Chrysanthemum indicum* L.	GB	Tashev (1962)
		*Dendranthema* sp.	GE	27.05.2009
			GV	28.10.2008
			R1	23.07.2010
				29.05.2009
			S1	20.10.2010
			S4	12.07.2011
			S5	07.10.2010
				18.12.2008
				26.05.2010
				28.04.2010
			S6	01.11.2011
				26.11.2009
*Macrosiphum (Macrosiphum) euphorbiae* (Thomas, 1878)	Acanthaceae	*Aphelandra squarrosa* Nees	R2	01.06.2010
	Anthericaceae	*Chlorophytum comosum* (Thunb.) Jacques	R2	01.06.2010
	Apocynaceae	*Mandevilla sanderii* (Hemsl.) Woodson	R2	01.06.2010
		*Vinca major* L.	R1	29.05.2009
	Araliaceae	*Schefflera arboricola* (Hayata) Merr.	R1	01.06.2010
	Asteraceae	*Cineraria* sp.	GB	Tashev (1962)
	Hydrangeaceae	*Hydrangea hortensis* Sm.	GB	Tashev (1962)
	Malvaceae	*Hibiscus rosa-sinensis* L.	GL	28.03.2011
				30.06.2009
			R2	01.06.2010
*Macrosiphum (Macrosiphum) rosae* (Linnaeus, 1758)	Rosaceae	*Rosa hybrida* Vill.	GE	27.05.2009
		*Rosa rugosa* Thunb.	GB	05.11.2010
*Myzus (Myzus) ornatus* Laing, 1932	Acanthaceae	*Acanthus* sp.	GB	Tashev (1962)
		*Fittonia argyroneura* E. Coem.	GB	Tashev (1962)
		*Ruellia speciosa* Lindau	GB	Tashev (1962)
	Amaranthaceae	*Iresine herbstii* Hook.	GV	25.05.2009
	Araliaceae	*Aralia sieboldii* Hort. ex K. Koch	GB	Tashev (1962)
*Myzus (Myzus) ornatus* Laing, 1932	Asparagaceae	*Asparagus* sp.	GB	Tashev (1962)
	Asteraceae	*Centaurea macrocephala* Muss. Pushk. ex Willd.	GB	Tashev (1962)
		*Chrysanthemum indicum* L.	GB	Tashev (1962)
		*Cineraria* sp.	GB	Tashev (1962)
	Begoniaceae	*Begonia* sp.	GB	Tashev (1962)
	Brassicaceae	*Arabis alpina* L.	GB	Tashev (1962)
	Ericaceae	*Erica australis* L.	GB	Tashev (1962)
		*Erica lusitanica* Rudolphi	GB	Tashev (1962)
		*Erica* sp.	GB	Tashev (1962)
	Fabaceae	*Lupinus* sp.	GB	Tashev (1962)
	Gesneriaceae	*Saintpaulia ionantha* H. Wendl.	GB	Tashev (1962)
	Hydrangeaceae	*Hydrangea hortensis* Sm.	GB	Tashev (1962)
	Lamiaceae	*Coleus x hybridus* Hort.	GV	25.05.2009
			GB	Tashev (1962)
	Mimosaceae	*Acacia floribunda* Willd.	GB	Tashev (1962)
	Oxalidaceae	*Oxalis floribunda* Lehm.	GB	Tashev (1962)
	Primulaceae	*Primula* sp.	GB	Tashev (1962)
	Saxifragaceae	*Heuchera* sp.	GB	Tashev (1962)
	Scrophulariaceae	*Digitalis purpurea* L.	GB	Tashev (1962)
	Urticaceae	*Laportea gigas* Wedd.	GB	Tashev (1962)
	Valerianaceae	*Valeriana montana* L.	GB	Tashev (1962)
	Violaceae	*Viola* sp.	GB	Tashev (1962)
*Myzus (Nectarosiphon) ascalonicus* Doncaster, 1946	Cucurbitaceae	*Lagenaria vulgaris* Ser.	GB	Tashev (1962)
	Geraniaceae	*Pelargonium* sp.	GB	Tashev (1962)
	Hydrangeaceae	*Hydrangea hortensis* Sm.	GB	Tashev (1962)
	Lamiaceae	*Salvia* sp.	GB	Tashev (1962)
*Myzus (Nectarosiphon) ascalonicus* Doncaster, 1946	Malvaceae	*Hibiscus rosa-sinensis* L.	GL	28.03.2011
	Resedaceae	*Reseda odorata* L.	GB	Tashev (1962)
	Scrophulariaceae	*Hebe* sp.	S1	01.07.2010
*Myzus (Nectarosiphon) persicae* (Sulzer, 1776)	Acanthaceae	*Aphelandra aurantiaca* Lindl.	GB	07.10.2010
		*Thunbergia coccinea* Wall.	GB	09.03.2009
				20.12.2008
	Agavaceae	*Cordyline terminalis* Kunth.	GS	05.03.2010
	Aizoaceae	*Aptenia cordifolia* (L.f.) Schwantes	GL	24.01.2011
	Amaranthaceae	*Pleuropetalum darwinii* Hook. f.	GS	17.03.2009
	Anthericaceae	*Chlorophytum comosum* (Thunb.) Jacques	GK	31.05.2010
			GL	11.07.2011
	Apocynaceae	*Catharanthus* sp.	GL	28.03.2011
		*Mandevilla sanderii* (Hemsl.) Woodson	S5	01.07.2010
				03.08.2010
				26.05.2010
	Araceae	*Anthurium andraeanum* Linden	V2	14.07.2009
		*Syngonium podophyllum* Schott	GL	24.01.2011
		*Zantedeschia aethiopica* (L.) Spreng.	GV	28.10.2008
	Araliaceae	*Hedera helix* L.	GL	14.03.2012
		*Schefflera arboricola* (Hayata) Merr.	GB	02.09.2010
				04.08.2010
				07.10.2010
			R1	01.06.2010
	Asteraceae	*Bellis perennis* L.	GB	20.10.2010
		*Chrysanthemum hybridum* Guss.	S1	13.03.2009
		*Chrysanthemum indicum* L.	GB	Tashev (1962)
		*Cineraria* sp.	GB	Tashev (1962)
		*Dendranthema* sp.	S1	13.03.2009
		*Gazania heterophylla* Willd. ex Steud.	S4	26.05.2010
		*Senecio hybridus* Scheidw.	S1	13.03.2009
		*Senecio rowleyanus* H. Jacobsen	GB	18.12.2008
		*Zinnia elegans* Jacq.	GE	01.06.2010
	Bignoniaceae	*Campsis radicans* (L.) Seem.	S5	30.08.2010
	Bombacaceae	*Ceiba pentandra* Gaertn.	GL	14.03.2012
	Brassicaceae	*Arabis alpina* L.	GB	20.10.2010
			GL	24.01.2011
	Cactaceae	*Zygocactus truncatus* K.Schum.	GS	05.03.2010
	Caprifoliaceae	*Viburnum tinus* L.	GB	02.09.2010
				07.10.2010
	Caryophyllaceae	*Dianthus hybridus* Schmidt ex Tausch	S6	31.05.2011
*Myzus (Nectarosiphon) persicae* (Sulzer, 1776)	Convolvulaceae	*Dichondra repens* J. R. Forst & G. Forst	S4	01.07.2010
				26.05.2010
				28.04.2010
		*Ipomoea purpurea* (L.) Roth	S4	28.04.2010
	Droseraceae	*Dionea* sp.	S3	06.08.2010
	Gesneriaceae	*Aeschynanthus radicans* Jack	GB	05.03.2010
	Hydrangeaceae	*Hydrangea hortensis* Sm.	GB	Tashev (1962)
	Lamiaceae	*Thymus* sp.	GB	05.11.2010
	Malvaceae	*Hibiscus rosa-sinensis* L.	GB	07.10.2010
				09.03.2009
			GL	24.01.2011
			S6	04.11.2009
		*Hibiscus* sp.	GS	17.12.2008
		*Hibiscus syriacus* L.	GL	28.05.2011
	Nyctaginaceae	*Bougainvillea glabra* Choisy	GB	04.08.2010
			R1	01.06.2010
			S4	28.04.2010
	Oleaceae	*Jasminum officinale* L.	GB	09.03.2009
	Primulaceae	*Cyclamen persicum* Mill.	S1	13.03.2009
	Solanaceae	*Calibrachoa* sp.	S5	26.05.2010
		*Datura hybrida* Ten.	GE	27.05.2009
		*Solandra maxima* (Sessè & Moc.) P. S. Green	GB	04.08.2010
				05.03.2010
				07.10.2010
		*Solandra* sp.	GB	26.05.2010
		*Solanum* sp.	GB	26.05.2010
	Zamiaceae	*Zamia pumila* L.	GB	18.12.2008
*Ovatus (Ovatus) crataegarius* (Walker, 1850)	Lamiaceae	*Monarda didyma* L.	R1	01.06.2010
*Periphyllus californiensis* (Shinji, 1917)	Aceraceae	*Acer palmatum* Thunb.	GB	20.10.2010
*Prociphilus (Meliarhizophagus) fraxinifolii* (Riley in Riley & Monell, 1879)	Oleaceae	*Fraxinus* sp.	R1	01.06.2010
*Prociphilus* sp.	Oleaceae	*Fraxinus excelsior* L.	GL	17.02. 2009
*Rhopalosiphum nymphaeae* (Linnaeus, 1761)	Nymphaeaceae	*Nymphaea alba* L.	GV	28.10.2008
*Rhopalosiphum padi* (Linnaeus, 1758)	Asphodelaceae	*Kniphophia uvaria* (L.) Hook.	R1	29.05.2009
	Poaceae	*Agrostis stolonifera* L.	S4	18.11.2010
		*Festuca ovina* L. subsp. *glauca* (Vill.) O. Bolòs & Vigo	R1	29.05.2009
*Tinocallis (Sarucallis) kahawaluokalani* (Kirkaldy, 1907)	Lythraceae	*Lagerstroemia indica* L.	R1	01.06.2010
				29.05.2009

* The specimens of *Aphis (Aphis) fabae* are not identified to the subspecies level.

## Discussion

Twenty three of all recorded aphid species (69.7 %) have been reported on indoor ornamental plants (Weiss 1916, Cichocka and Goszczynski 1975, Achremowicz et al. 1986, Cichocka 1992, Łabanowski 2008, Rafi et al. 2010) and only six (18.2 %) have been found on ornamentals in Bulgarian greenhouses (Tashev 1962).

**Figure 1.**
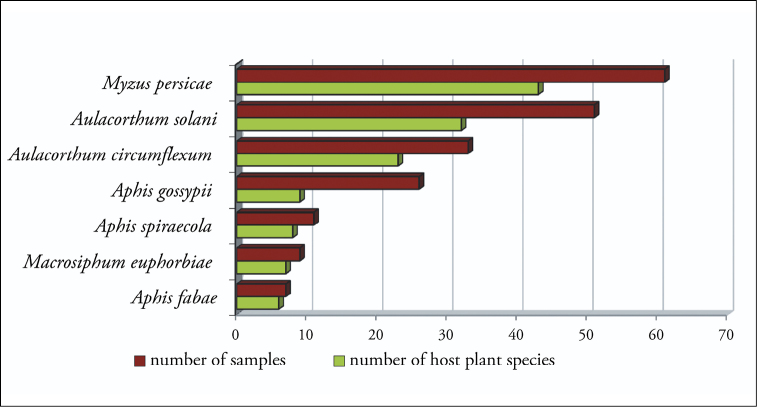
Number of samples and number of host plant species found by polyphagous aphid species.

Twenty one of all recorded aphid species are polyphagous, 9 are oligophagous and only one is monophagous (Blackman and Eastop 1994, 2000, 2006). Seven of the polyphagous species were observed more frequently (Fig. 1).

During the survey, the most common species, represented by the highest numbers of samples, was *Myzus persicae*, found throughout the year in 13 greenhouses (61 samples, 21.9 %) (Fig. 1), followed by *Aulacorthum solani* (10 greenhouses, 51 samples, 18.3 %), *Aulacorthum circumflexum* (3 greenhouses, 33 samples, 11.8 %), *Aphis gossypii* (9 greenhouses, 26 samples, 9.3 %), *Aphis spiraecola* (4 greenhouses, 11 samples, 3.9 %), *Macrosiphum euphorbiae* (3 greenhouses, 9 samples, 3.2 %) and *Aphis fabae* (3 greenhouses, 8 samples, 2.9 %). All other species were found very rarely and were represented by 5 or less samples (< 1.8 %).

**Figure 2.**
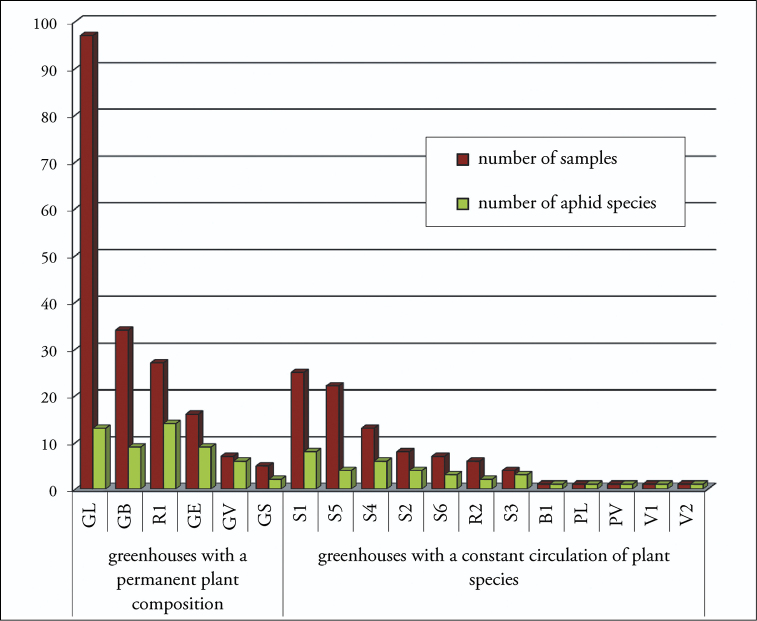
Distribution of number of samples and number of aphid species by greenhouses.

The present study indicates that in greenhouses with a permanent plant composition, aphid infestations are more frequent, more widespread, and are caused by a greater variety of species compared to infestations in greenhouses with a constant circulation of plant species (Fig. 2).

The widest host range was shown by *Myzus persicae* (43 hosts, 38 %), *Aulacorthum solani* (32 hosts, 28 %), *Aulacorthum circumflexum* (23 hosts, 20 %), *Aphis gossypii* (9 hosts, 8 %), *Aphis spiraecola* (8 hosts, 7 %), *Aphis fabae* (7 hosts, 6 %) and *Macrosiphum euphorbiae* (6 hosts, 5 %).

*Periphyllus californiensis* and *Aphis (Aphis) fabae mordvilkoi* are reported for the first time for Bulgaria. Furthermore, *Aphis spiraecola* has been found in new localities and has widened its host range in this country. So far, this species had been reported only on apple by Rasheva and Andreev (2007). All hosts reported in the present study for *Aphis spiraecola* are new for Bulgaria.

The list of host plants includes 114 species from 95 genera and 58 families. The most frequently infested plant species belong to Asteraceae (50 samples) and Malvaceae (37 samples). Eighteen samples were collected from Araceae; 16 samples from Apocynaceae; 15 samples from Araliaceae; 13 samples from Geraniaceae; and 10 samples from Acanthaceae, Solanaceae and Rosaceae.

The most frequently infested plants belong to the genera *Hibiscus* (12.9 %, 36 samples), *Dendranthema* (6.8 %, 19 samples), *Chrysanthemum* (5 %, 14 samples) and *Pelargonium* (4.7 %, 13 samples).

The highest diversity of aphid species was observed on *Hibiscus rosa-sinensis* and consists of 9 species (from 29 samples). Five species were identified on *Dendranthema* sp. (19 samples), *Chrysanthemum hybridum* (13 samples) and *Anthurium andreanum* (6 samples). Four species were found on *Cyclamen persicum*, *Shefflera arboricola* and *Syngonium podophyllum*. Three species were recorded on *Aptenia cordifolia*, *Bougainvillea glabra*, *Chlorophytum comosum*, *Hebe* sp., *Hedera helix*, *Hibiscus syriacus*, *Mandevilla sanderi* and *Vinca major*.

## Conclusion

The aphids established on ornamental plants in greenhouses in Bulgaria comprise 33 species and one subspecies from 13 genera and 5 subfamilies.The most widespread aphid species is *Myzus persicae*, followed by *Aulacorthum solani* and *Aphis gossypii*. The widest host ranges were shown by *Myzus persicae*, *Aulacorthum solani* and *Aulacorthum circumflexum*.

The list of host plants includes 114 species from 95 genera and 58 families. The most infested plants belong to the genera *Hibiscus* and *Dendranthema*. The largest number of aphid species was detected on *Hibiscus* (9 species).
